# Establishing key criteria to define and compare models of specialist palliative care: A mixed-methods study using qualitative interviews and Delphi survey

**DOI:** 10.1177/0269216319858237

**Published:** 2019-06-28

**Authors:** Alice M Firth, Suzanne M O’Brien, Ping Guo, Jane Seymour, Heather Richardson, Christopher Bridges, Mevhibe B Hocaoglu, Gunn Grande, Mendwas Dzingina, Irene J Higginson, Fliss EM Murtagh

**Affiliations:** 1 Cicely Saunders Institute of Palliative Care, Policy and Rehabilitation, King’s College London, London, UK; 2 School of Nursing and Midwifery, The University of Sheffield, Sheffield, UK; 3 St. Christopher’s Hospice, Sydenham, UK; 4 Palliative Care Team, King’s College Hospital NHS Foundation Trust, London, UK; 5 Faculty of Arts & Sciences, Department of Psychology, Eastern Mediterranean University, Famagusta, Cyprus; 6 Dr Fazil Kucuk Faculty of Medicine, Eastern Mediterranean University, Famagusta, Cyprus; 7 Division of Nursing, Midwifery & Social Work, School of Health Sciences, The University of Manchester, Manchester, UK; 8 Wolfson Palliative Care Research Centre, Hull York Medical School, University of Hull, Hull, UK

**Keywords:** palliative care, Delphi technique, models, organisational, hospices

## Abstract

**Background:**

Specialist palliative care services have various configurations of staff, processes and interventions, which determine how care is delivered. Currently, there is no consistent way to define and distinguish these different models of care.

**Aim:**

To identify the core components that characterise and differentiate existing models of specialist palliative care in the United Kingdom.

**Design:**

Mixed-methods study: (1) semi-structured interviews to identify criteria, (2) two-round Delphi study to rank/refine criteria, and (3) structured interviews to test/refine criteria.

**Setting/participants:**

Specialist palliative care stakeholders from hospice inpatient, hospital advisory, and community settings.

**Results:**

(1) *Semi-structured interviews* with 14 clinical leads, from eight UK organisations (five hospice inpatient units, two hospital advisory teams, five community teams), provided 34 preliminary criteria.
(2) *Delphi study*: Round 1 (54 participants): thirty-four criteria presented, seven removed and seven added. Round 2 (30 participants): these 34 criteria were ranked with the 15 highest ranked criteria, including setting, type of care, size of service, diagnoses, disciplines, mode of care, types of interventions, ‘out-of-hours’ components (referrals, times, disciplines, mode of care, type of care), external education, use of measures, bereavement follow-up and complex grief provision.
(3) *Structured interviews* with 21 UK service leads (six hospice inpatients, four hospital advisory and nine community teams) refined the criteria from (1) and (2), and provided four further contextual criteria (team purpose, funding, self-referral acceptance and discharge).

**Conclusion:**

In this innovative study, we derive 20 criteria to characterise and differentiate models of specialist palliative care – a major paradigm shift to enable accurate reporting and comparison in practice and research.

**What is already known about the topic?**

- Specialist palliative care is facing an increasing, ageing population and restricted resources.
- Currently, there is no consistency in the way models of specialist palliative care are defined in clinical practice or research.
- This constrains our understanding of what models of care (or components) are most clinically effective and cost-effective.

**What this paper adds?**

- This paper provides a set of criteria to define and compare models of UK specialist palliative care.

**Implications for practice, theory or policy**

- Researchers and clinicians will be able to clearly define and distinguish models of specialist palliative care.

## Background

Specialist palliative care is facing an increasing, ageing population and restricted resources.^1^ If recent mortality trends continue, 160,000 more people in England and Wales will need palliative care by 2040,^1^ and healthcare systems and models of specialist palliative care will need to adapt to meet the rapidly growing needs of palliative care. Existing models of specialist palliative care are often historically oriented towards cancer care and may lack responsiveness to societies’ changing needs.^2^ There are also major geographical variations in NHS provision of care resulting in often poor match between palliative care needs of patients and families and the resources provided to meet these needs.^3,4^

To improve responsiveness of specialist palliative care and evolve models of care to better meet population needs, we need to understand and define different models of care. The term ‘model of care’ is used infrequently and inconsistently in the published evidence on specialist palliative care.^5^ A ‘model of care’ has been defined as the way in which health care services are delivered and is ‘a descriptive picture of practice which adequately represents the real thing’.^6^

While there is agreement on the definition of specialist palliative care,^7^ existing models of specialist palliative care are not characterised or reported in a consistent way. This limits the ability to compare and evaluate existing or new models. The underreporting of the components of specialist palliative care services and the inability to compare and contrast different models are well recognised^8,9^ and are major barriers to the evolution of specialist palliative care.^9^ Once models are consistently defined, comparisons between models can more readily be made, and research can be conducted into which components of a model of care increase effectiveness and cost-effectiveness.

Specialist palliative care services are provided in a range of different settings, including hospital, home, hospice inpatient units, outpatients and day services.^10^ Current research largely focuses on the effectiveness of palliative care in a specific setting (hospice inpatient, hospital or community) or in the specialty as a whole.^11–13^ However, specialist palliative care even within one setting (hospital, hospice or home) is delivered in a wide range of different ways.^5,14–16^ Therefore, studies on effectiveness of one setting or the profession as a whole include diverse teams that provide specialist palliative care in differing ways, including offering a variety of interventions, skill mix, the patient population they see and frequencies of visit.^12^ More work is needed to test the specific components of palliative care team activity and to discover which configurations or components are most clinically effective and cost-effective.^12^ It is therefore important that specialist palliative care services can consistently define their models of care,^5^ to develop the foundational work that will allow for comparisons between models and will enable further research into effectiveness of different models.^12^

We therefore aimed to identify the core components that characterise and differentiate existing models of specialist palliative care in the United Kingdom.

## Methods

This study employed a mixed-methods design and was conducted in three stages: (1) semi-structured interviews to derive criteria from a range of established and innovative existing models of UK specialist palliative care, (2) Delphi study with expert consensus to identify any missing criteria, refine the criteria derived from Stage 1 and rank them in terms of overall importance, and (3) structured interviews with hospice inpatient, hospital advisory, and community team leads to test and refine the criteria derived from Stages 1 and 2. This study was UK based and may not apply to other countries, although it could provide preliminary criteria as a basis for a similar study elsewhere.

### Stage 1: semi-structured interviews

A rapid scoping review (Supplemental Appendix 5) was conducted to identify literature related to models of palliative care. Original papers and reviews were examined for possible criteria that could help define models of specialist palliative care and a topic guide was created covering the 28 preliminary criteria identified from this literature (Supplemental Appendix 1). Semi-structured interviews using the topic guide (Supplemental Appendix 1) were conducted with 14 palliative care service or team leads from eight organisations discussing 12 settings of care (five hospice inpatient units, two hospital advisory teams, and five community teams). These organisations were taking part in a programme of research – C-CHANGE – that aims to develop and validate a case-mix classification for palliative care in the United Kingdom (funded by the National Institute for Health Research (NIHR) RP-PG-1210-12015). These organisations had been selected to be nationally representative in terms of the populations served. Participants consented to be interviewed and recorded. The interviews were begun by asking participants whether they could describe how care was provided in their own service(s) (Supplemental Appendix 1, Prompt 4). The questions on the interview guide were used as prompts. Audio recordings of the interviews were analysed, using thematic content analysis to identify all the criteria that were discussed in the interviews to characterise the various models of specialist palliative care. Two researchers (A.F. and S.O’B.) independently analysed the interviews, results were compared and – where there was disagreement – discussed with a third researcher (F.M.) until consensus was reached. These criteria were then used for Stage 2.

### Stage 2: Delphi study

We selected the Delphi survey method for this second stage as it enabled us to present potential criteria derived from Stage 1 to all respondents, allowed them time to absorb this complex information at their own speed and enabled us to sample a wide range of views in a way which allowed for all opinions to have equal weight. A two-round Delphi survey of UK clinical, policy or patient/public involvement leads were invited from the OACC (The Outcome Assessment and Complexity Collaborative) network (a multidisciplinary network of professionals engaged in the implementation of outcome measures in specialist palliative care in England – see www.kcl.ac.uk/nursing/departments/cicelysaunders/research/studies/oacc/index.aspx), and the national NIHR-funded project C-CHANGE sites (RP-PG-1210-12015). Participants were told that we were aiming to establish a list of key criteria to describe and compare models of care. The Delphi study was conducted to refine the criteria from Stage 1, to identify any additional criteria, to achieve consensus on how each criterion was defined and to rank the criteria in terms of importance. The Delphi method was chosen as it is a widely known method for group decision making,^17^ allowing for a range of views without undue dominance from any participants (important when the status and seniority of participants are varied). CREDES (Guidance on Conducting and REporting DElphi Studies) in palliative care were followed.^18^

An online survey was developed using Bristol Online Survey.^19^ The survey was piloted for face validity by four palliative care clinicians (non-participants) prior to the survey going live. Email invitations were sent out and participants received a description of the Delphi study and instructions on how to access the online survey. Consent was assumed for any participant who chose to reply to the survey. Each round of the survey remained open for 2½ weeks and one reminder email was sent for each round of the survey a week before the survey closed.

#### Delphi Round 1: evaluation of preliminary criteria

In the first Delphi round, panel members were presented with a list of 34 criteria from Stage 1. Participants were asked to state whether they agreed with the inclusion of each criterion as important for describing and comparing models of specialist palliative care (yes/no/don’t know) and their reasons for this. Participants were advised that we aimed to reduce the list of components to those that were most useful to characterise and compare models. They were also asked to comment on the phrasing and clarity of the criterion, as well as the answer options listed. Finally, participants were asked to suggest any additional criteria they thought should be included.

Responses were analysed and collated, and each criterion was retained if at least 75% participants agreed ‘Yes’. Data were collated and analysed using IBM SPSS version 22 using descriptive analysis (frequencies). Free-text comments were analysed using content analysis and used to refine and expand the set of criteria.

#### Delphi Round 2: feedback and ranking

In Round 2 of the Delphi process, participants received anonymised feedback from Round 1 and the amended list of criteria for further refinement and ranking. Participants were asked to rate the importance of each criterion for characterising and comparing different models of care on a 5-point Likert-type scale (1 = not at all important; 2 = not very important; 3 = important; 4 = very important; 5 = extremely important). In addition to the rating scales, participants were also given the opportunity to add additional free-text comments to help refine criterion and answer options.

Responses were analysed to capture both central tendency (median rating) and dispersion (interquartile range (IQR)). Consensus was deemed to have been reached for criteria that received aggregated responses with an IQR of ⩽1 and a median of 4 or 5. Both methods are considered to offer robust measurements for Delphi surveys.^20,21^ Criteria reaching this consensus were then included in the final set.

Ranking responses were collated and analysed using IBM SPSS. Free-text responses underwent content analysis and were used to refine the criteria and response options.

#### Stage 3: structured interviews to test for acceptability and feasibility

The criteria developed from Stage 2 were then tested with clinical leads from three different specialist palliative care settings (hospice inpatient, hospital advisory teams and community) settings, using structured interviews. These were the same organisations taking part in the C-CHANGE programme of research (funded by the NIHR RP-PG-1210-12015) that had participated in Stage 1, although 18 months passed between conducting Stage 1 and Stage 3 interviews, and some leads and services (especially in community settings) had changed. Participants consented to be interviewed and recorded. Results from these interviews were entered into Excel to identify whether criteria were able to discriminate between services.

### Ethics

Ethical approval was received from King’s College London (LRS-15/16-2449).

## Results

### Stage 1: semi-structured interviews

Semi-structured interviews were conducted with 14 service leads, from eight organisations, discussing 12 settings of care (five hospice inpatient units, two hospital advisory teams and five community teams). Interviews were median 72 min (range = 48–101 min).

**Figure 1.**
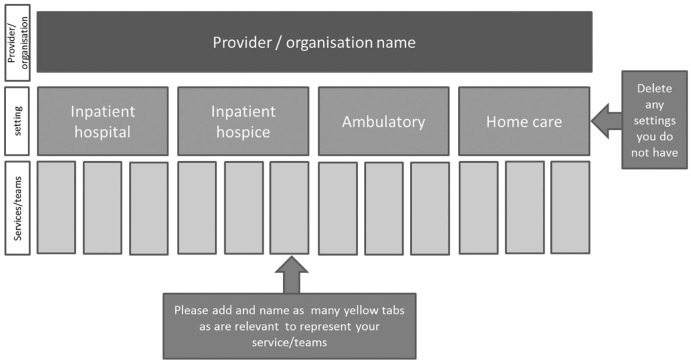
Defining multiple models of specialist palliative care within one organisation.

An early finding was that the clinical leads struggled to know at which level within the organisation to describe their models of care: ‘Sorry, which bit of our service do you want us to describe? There’s so much of it here and it’s all run quite differently’ (Interview 3). It was often confusing when an organisation covered multiple settings of care (i.e. hospice inpatient, community, hospital inpatient, day services) and also provided multiple services within each setting, which often overlapped. For example, a hospice may have inpatient hospice, homecare and ambulatory settings. Within any one of these settings, multiple services or teams were often running. Within the day services, there may be a physiotherapy clinic, a lymphoedema service, and a day service, all operating with different models of care. Figure 1 was therefore developed after the first three interviews to help facilitate understanding and guide subsequent interviews.

After all interviews were completed, from the 28 criteria in the topic guide, 11 were removed as not reported as useful; 17 criteria were refined; and a further 17 criteria were created. This resulted in 34 criteria to take forward into Stage 2 (see Table 2).

### Stage 2: Delphi survey

#### Delphi Round 1 (assessing eligibility of criteria)

A total of 190 participants were invited to take part in the Delphi survey. Of the 190 clinical, policy and patient/public involvement leads contacted, 54 agreed to participate (response rate = 28.4%). Demographic details of participants who took part in Delphi Rounds 1 and 2 are shown in Table 1.

**Table 1.** Demographic information for Delphi Round 1 and Round 2 respondents.

	Round 1 (n = 54)	Round 2 (n = 30)
Staff group		
Palliative care consultant	19	11
Palliative care registrar	3	2
Palliative care clinical nurse specialist	8	4
Other nurse	4	2
Other healthcare professional	1	1
Occupational therapist	1	1
Team lead	3	4
Other	13	5
Patient, family or public representative	2	0
Primary setting of work		
Hospital inpatient	6	0
Hospice outpatient	0	1
Hospice community	3	2
Hospital advisory	10	7
Community	4	0
Works across multiple settings	20	9
Other	11	11
Palliative care experience (in years)		
<2	0	0
2–4	5	3
4–8	7	3
>8	39	21
Not applicable/missing	3	3

##### Removal of seven criteria

Results of Round 1 are presented in Table 2; of 34 criteria, six were removed due to not reaching the 75% consensus rate (Criteria 3, 9, 11, 14, 17 and 34). After analysing respondents’ free-text comments, it was further agreed that Criterion 10 (multidisciplinary team meetings) would also be removed (five participants had interpreted the criterion differently, and two further participants had not understood it. Multidisciplinary team meetings/discussions were also reported by some respondents as ‘standard’ for all specialist palliative care teams, and it was felt this criterion would not therefore discriminate between different models of care).

**Table 2.** Delphi results.

Delphi Round 1 results			
Criteria which reached consensus rate of 75% (n = 54)			
	Summary of criterion	% of participants in agreement	Reached consensus rate of 75%
Criterion 1	Setting of care	94.3	Yes
Criterion 2	Hands on or advisory	84.9	Yes
Criterion 3	Referral route	64.2	No
Criterion 4	Standardised referral criteria	81.1	Yes
Criterion 5	Referrals on urgency	83	Yes
Criterion 6	Medical responsibility	86.8	Yes
Criterion 7	Discharge criteria	83	Yes
Criterion 8	Number of disciplines	98.1	Yes
Criterion 9	Key worker	73.6	No
Criterion 10	MDT meetings	84.9	Yes, but removed due to free-text comments which showed a range of interpretations/confusion around the criterion.
Criterion 11	Disease specific or comprehensive	73.6	No, but refined and added back into R2 due to free-text comments and research team discussion
Criterion 12	Purpose of service	94.3	Yes
Criterion 13	Mode	92.5	Yes
Criterion 14	Frequency of care	73.6	No
Criterion 15	Time limited	77.4	Yes
Criterion 16	Interventions	90.6	Yes
Criterion 17	Transport	64.2	No
Criterion 18	Out-of-hours referrals	86.8	Yes
Criterion 19	Out-of-hours provision	92.5	Yes
Criterion 20	Out-of-hours availability	92.5	Yes
Criterion 21	Out-of-hours mode	92.5	Yes
Criterion 22	Out-of-hours hands on or advisory	88.7	Yes
Criterion 23	Out-of-hours disciplines	94.3	Yes
Criterion 24	Education and training	86.8	Yes
Criterion 25	Coordination systems	84.9	Yes
Criterion 26	Outcomes	84.9	Yes
Criterion 27	Experience measures	79.2	Yes
Criterion 28	Post-death follow-up	94.3	Yes
Criterion 29	Complex grief assessment	86.8	Yes
Criterion 30	Bereavement risk assessment	79.2	Yes
Criterion 31	Number of disciplines providing bereavement support	86.8	Yes
Criterion 32	Offering external bereavement support	92.5	Yes
Criterion 33	Bereavement care time frame	83	Yes
Criterion 34	Geographical bases	71.7	No
Delphi Round 2 results			
Criteria which reached consensus rate of IQR ⩽1 and median >4 (n = 30)			
Summary of criterion		Median	IQR
^a^Setting of care (inpatient hospital, inpatient hospice, home based, etc.)		5	1
^a^Type of care (‘hands on’, advice or education)		5	1
^b^Referrals accepted annually		4	1
Number of disciplines		4	1
^a^Mode of care		4	1
^a^Intervention available		4	1
‘Out-of-hours’ referrals		5	1
^a^‘Out-of-hours’ available to patients already known		4.5	1
^a^‘Out-of-hours’ availability		4.5	1
‘Out-of-hours’ mode		4	1
^a^Type of ‘out-of-hours’ provision		4	1
Education and Training		4	1
Experience measures		4	1
Post-death follow-up		4	1
^a^Complex grief assessment		4	1
^a^Primary diagnosis		4	1
Criteria removed due to not reaching consensus rate			
Standard criteria for accepting referrals		4	2
^a^Priority of referrals		4	2
^b^Self-referrals		3	2
Primary medical responsibility		4	2
Discharge criteria		3.5	1
^b^First assessment		3	1
^a^Comprehensive care or specific symptom or disease-related interventions		4	2
Purpose of care		4	2
^a^Time limited		3	2
^a^‘Out-of-hours’ disciplines		4	2
^a^Bereavement risk assessment		4	2
Offering bereavement care to general public		4	2
^a^Bereavement risk evaluation		3.5	1
Number of disciplines providing bereavement support		3	2
^a^Offering bereavement care to general public		3.5	1
Time period for complex grief assessment		3	1
^a^Age of patients		4	2
^a^Public or voluntary		4	2

IQR, interquartile range; MDT, multidisciplinary team.

a Refined criteria from previous round.

b New criteria based on feedback from Round 1.

##### Addition of seven new criteria

After reviewing responses to the final question of the survey, ‘*Do you think there are any criteria that we have not included?*’, three new criteria were added to the list based on suggestions from the experts. These included the following: (1) *How many referrals are accepted and seen annually by this service/team?* (2) *Does this service/team accept patient or family self-referrals?* (3) *Who undertakes the first assessment?* The out-of-hours criteria were heavily refined to improve comprehension and four new criteria relating to ‘out-of-hours’ were created. This resulted in a refined list of 34 criteria.

#### Delphi Round 2 (ranking of criteria)

Thirty participants (of 54 in Round 1) completed Round 2 (60% response rate). The 34 revised criteria were ranked and rated, and criteria not meeting the predetermined consensus level were excluded. Sixteen criteria reached consensus (Table 3). These 16 criteria included setting, type of care, size of service, diagnosis, disciplines, mode of care, types of interventions, out-of-hours referrals, out-of-hours service times, disciplines of out-of-hours care, mode of out-of-hours care, type of out-of-hours care, external education, outcomes and experience measures, standard bereavement follow-up and provision for complex grief.

**Table 3.** Final agreed criteria to define models of palliative care.

Sixteen criteria which reached consensus on Rounds 1 and 2 of Delphi survey	
1	Setting of care (inpatient hospital, inpatient hospice, home based, etc.)
2	Type of care delivered (‘hands on’ or advisory)
3	Size – measured by number of referrals accepted annually
4	Number of disciplines delivering the care
5	Mode of care (‘face to face’, telephone, or other remote delivery)
6	Number of interventions available
7	Whether ‘out-of-hours’ referrals are accepted
8	Whether ‘out-of-hours’ care is available to patients already known to the service
9	Time when is ‘out-of-hours’ care available?
10	‘Out-of-hours’ mode (‘face to face’ or advisory)
11	Type of ‘Out-of-hours’ provision (‘hands on’ or advisory)
12	Extent of education/training provided to external professionals
13	Whether outcome and experience measures are used in the service
14	Whether standard bereavement follow-up is provided?
15	Whether complex grief follow-up is provided?
16	The primary diagnosis of those patients receiving care (cancer/non-cancer)
Four further criteria included into final set following testing/feedback from structured interviews	
17	Is service a publicly funded or voluntary funded service?
18	Patient or family self-referrals or not?
19	Whether there are standard discharge criteria?
20	Purpose of care provided

### Stage 3: structured interviews

Interviews were conducted with 21 service leads from 19 different services (six hospice inpatients, four hospital advisory and nine community settings). The responses to each criterion were compared to see whether the criteria could distinguish and discriminate effectively between services (see Tables 4 and 5). A further four criteria relating to context were also added; these were felt to be important by the clinical leads for the practical application of the criteria and to ensure a more thorough representation of the context for each model of care. These four ‘contextual criteria’ were the purpose of the team, who funds/manages the team, ability to self-refer, and discharge of patients (Supplemental Appendix 2).

**Table 4.** Comparing services models of care Criteria 1–9.

Service identifier	C1. Setting	C2. Hands on or advisory	C3. Size of service (annual referrals)	C4. Number of disciplines (see Supplemental Appendix)	C5. Mode of care	C6. Number of interventions available	C7. Can referrals be made and seen OOH?	C8A. OOH medical availability	C8B. OOH nursing availability	C9. OOH service availability
A	Home-based care	Advisory care	Medium	3	Combination	1	No	None	On call	High
B	Combination	Advisory care	Small	2	Combination	0	No	None	On call	High
C	Home-based care	Advisory care	Large	13	Combination	1	Yes	On call	Resident	Low
D	Combination	Combination	Medium	12	Combination	3	Yes	On call	Resident	Low
E	Outpatients	Combination	Small	2	Combination	11	No	None	None	None
F	Home-based care	Advisory care	Medium	3	Combination	2	No	On call	On call	High
G	Outpatients	Combination	Small	2	Combination	2	No	None	On call	High
H	Home-based care	Combination	Large	13	Combination	1	Yes	On call	Resident	High
I	Home-based care	Advisory care	Medium	6	Combination	0	Yes	On call	On call	High
J	Inpatient hospital	Advisory care	Large	3	Combination	11	No	On call	None	On call
K	Inpatient hospital	Advisory care	Large	4	Combination	12	No	On call	None	On call
L	Inpatient hospital	Advisory care	Large	4	Combination	12	Yes	On call	On call	On call
M	Inpatient hospital	Advisory care	Large	5	Combination	11	Yes	On call	None	On call
N	Inpatient hospice	Combination	Medium	14	Face to face	7	Yes	On call	Resident	High
O	Inpatient hospice	Combination	Small	17	Face to face	12	Yes	No	Resident	High
P	Inpatient hospice	Combination	Small	16	Face to face	8	No	On call	Resident	High
Q	Inpatient hospice	Combination	Large	15	Combination	8	Yes	On call	Resident	High
R	Inpatient hospice	Combination	Medium	16	Combination	12	Yes	Resident	Resident	High
S	Inpatient hospice	Combination	Large	18	Face to face	9	Yes	On call	Resident	High

OOH, out of hours.

**Table 5.** Comparing services models of care Criteria 10–17.

Service identifier	C10. OOH mode	C11. OOH advisory or hands on	C12. External education provided	C13A. Outcomes	C13B. Experience	C14. Mode of bereavement follow-up	C15A. Provide complex grief follow-up for adult	C15B. Provide complex grief follow-up for children	C16. Diagnosis	C17A Service managed by	C17B. Service funded by
A	Combination	Combination	Yes	Yes	Yes	Medium	Yes	Yes	Any life-limiting illness	Voluntary/charitable	Both
B	Combination	Combination	Yes	Yes	Yes	Medium	Yes	Yes	Non-cancer only	Voluntary/charitable	Both
C	Combination	Combination	Yes	Yes	Yes	Medium	Yes	Yes	Any life-limiting illness	Voluntary/charitable	Both
D	Telephone	Advisory	Yes	Yes	Yes	?	yes	yes	Any life-limiting illness	Both	Both
E	NA	NA	No	No	No	None	No	Standard only	Any life-limiting illness	Statutory/public	Statutory/public
F	Telephone	Advisory	Yes	Yes	Yes	High	No	No	Any life-limiting illness	Statutory/public	Statutory/public
G	Telephone	Advisory	Yes	Yes	Yes	None	Yes	Yes	Any life-limiting illness	Statutory/public	Statutory/public
H	Combination	Combination	Yes	Yes	Yes	High	Yes	Yes	Any life-limiting illness	Voluntary/charitable	Both
I	Combination	Combination	Yes	No	Yes	High	Yes	No	Any life-limiting illness	Statutory/public	Statutory/public
J	Telephone	Advisory	No	Yes	Yes	None	None	None	Any life-limiting illness	Statutory/public	Statutory/public
K	Telephone	Advisory	No	Yes	No	Medium	Yes	Yes	Any life-limiting illness	Statutory/public	Statutory/public
L	Combination	Advisory	Yes	Yes	No	Medium	Yes	Yes	Any life-limiting illness	Statutory/public	Statutory/public
M	Combination	Combination	No	Yes	Yes	Low	Yes	Yes	Any life-limiting illness	Voluntary	Combination
N	Combination	Combination	Yes	Yes	Yes	High	Yes	Yes	Any life-limiting illness	Voluntary	Statutory/public
O	Face to face	Face to face	Yes	Yes	Yes	None	No	No	Any life-limiting illness	Combination	Combination
P	Combination	Combination	Yes	Yes	Yes	Low	Yes	No	Any life-limiting illness	Statutory/public	Statutory/public
Q	Combination	Combination	Yes	Yes	Yes	High	Yes	Yes	Any life-limiting illness	Statutory/public	Combination
R	Combination	Combination	Yes	Yes	Yes	Low	Yes	Yes	Any life-limiting illness	Voluntary	Combination
S	Combination	Combination	Yes	Yes	Yes	Low	Yes	Yes	Any life-limiting illness	Combination	Combination

OOH, out of hours.

## Discussion

This is, to the best of our knowledge, one of the first attempts at deriving empirical criteria that may be used to define and distinguish different specialist palliative care models. Using mixed methods, we have developed a set of criteria from these primary data to characterise and distinguish different UK specialist palliative care services (including setting, type of care, size of service, diagnoses accepted, disciplines, mode of care, types of interventions, out-of-hours characteristics, external education provision, use of outcome/experience measures, bereavement provision, plus the purpose of the team, who funds/manages the team, ability to self-refer, and discharge processes). These criteria capture the key *differentiating* components between different UK models of specialist palliative care across settings (hospice inpatients, hospital and community-based) and will – for the first time – enable these different models of care to be described and compared accurately for clinical and commissioning purposes. This study also provides the foundational work that will enable research to be conducted on which components of a model of care increase effectiveness and cost-effectiveness.

It is important to note, however, that these criteria should *not* be used to inform a ‘baseline’ level of specialist palliative care service; by the very nature of this study, we have identified criteria that *differentiate* between existing models. It follows, therefore, that – inevitably – some specialist palliative care services will provide some elements and not others; this is to be expected, given the purpose and methodology of our work. Other characteristics, such as holistic care, training in specialist palliative care and the use of multidisciplinary teams in delivery of care – are considered to be ‘core’ to the definition of specialist palliative care,^7^ so they are not included in these *differentiating* criteria.

Palliative care services until now have often been described simply in terms of their place of delivery, receiving care at hospice, hospital, ambulatory unit or in the community, with very limited description of their varying components. More detailed description of the models of care components are needed due to the large variation in service provision.^10^ Bainbridge et al.,^8^ in their review of systematic reviews of community end-of-life care, do report which components are most strongly associated with positive outcomes. Of note, core elements (a holistic care model, end-of-life training and multidisciplinary care) were most strongly associated with positive outcomes. However, this may simply reflect the limitations of the evidence, with models of care rarely reported in the evidence. Having criteria to define models of UK specialist palliative care will enable researchers to conduct evaluations of various models of care and compare the effectiveness and cost-effectiveness of different models to determine which elements work best (and most cost-effectively). It will also enable specialist palliative care services to clearly define their services and consider the similarities and differences between the services they offer and other providers.

The strengths of this study are that it has sought expert consensus from ‘real world’ professionals to identify the key criteria to characterise and differentiate these highly varied models of specialist palliative care. It has also used a sequential mixed-methods approach to painstakingly build a model framework – step-by-step – using empirical data. As recommended, we used the Delphi process to ‘explore or expose underlying assumptions or information’ and to ‘seek out information’ and ‘correlate informed judgements’.^17^ We also followed the recommendations of recent guidance on conducting and reporting Delphi studies in palliative care,^18^ including justification for our choice of Delphi, detail of the process, definition of how consensus was defined/reached, piloting, and reporting steps. We have not had external validation. Other limitations are that this study is UK based and may not apply to other countries (although it could provide preliminary criteria as a basis for a similar study elsewhere). The response rate to the Delphi study was low, and dropout for Round 2 of the Delphi was high; however, this was not surprising due to the participants being clinical staff and the considerable time and engagement required from participants to complete the Delphi.

## Conclusion

Until now, there has not been a clear set of criteria to define models of UK specialist palliative care, making it challenging to compare different models of care provided by services. This paper identifies 20 criteria to characterise and differentiate models of specialist palliative care – a major paradigm shift to enable accurate reporting and comparison in practice and research.

## Supplemental Material

858237_supp_mat – Supplemental material for Establishing key criteria to define and compare models of specialist palliative care: A mixed-methods study using qualitative interviews and Delphi survey Click here for additional data file. Supplemental material, 858237_supp_mat for Establishing key criteria to define and compare models of specialist palliative care: A mixed-methods study using qualitative interviews and Delphi survey by Alice M Firth, Suzanne M O’Brien, Ping Guo, Jane Seymour, Heather Richardson, Christopher Bridges, Mevhibe B Hocaoglu, Gunn Grande, Mendwas Dzingina, Irene J Higginson and Fliss EM Murtagh in Palliative Medicine
