# Supplementary material for: Establishing key criteria to define and compare models of specialist palliative care: A mixed-methods study using qualitative interviews and Delphi survey
Source: Palliat Med. 2019 Jun 28;33(8):1114–24. doi: 10.1177/0269216319858237 (PMC6691593; doi:10.1177/0269216319858237)
Supplement: 858237_supp_mat – Supplemental material for Establishing key criteria to define and compare models of specialist palliative care: A mixed-methods study using qualitative interviews and Delphi survey [file 858237_supp_mat.pdf]

Appendix 1: Stage 1 Topic guide

Name of setting:

Name of service:

Name of interviewee and job title:

	**Prompts for interview**	
1	What is the physical address/es of the (base) setting of care	Removed
2	What areas do your services cover?	Refined for Delphi
3	What is the label/description used by your teams that best portrays your team's delivery of care?	Removed
4	Can you describe the Model of Care in your………………….. setting? What are the essentials necessary for your model of care to be delivered?	Removed
5	What is the main focus of the services (what mainly and mostly the setting of care does and what it allocates most of its resources to doing.)<br>- Palliative day care<br>- Community Care (including Home Care and Hospice at Home, home support team)<br>- Hospice beds<br>- Palliative hospital support<br>- Inpatient<br>- Outpatient<br>- Palliative care outpatient clinic<br>- Bereavement support<br>- Other:	Refined for Delphi

6.	Primary condition patients present with	Refined for Delphi
7.	Advisory versus Hands on care?	Refined for Delphi
8.	What are the sources of funding for your institution (Private, voluntary, NHS, Other)?	Refined for Delphi
9.	Do you submit to the Minimum Data Set?	Removed
10.	Staff numbers and roles	Refined for Delphi
11.	What is the total number of employees this year?	Removed
12.	What is the total number of volunteers this financial year?	Removed
13.	1. Regarding coordination of care:<br>How do you do it? How do you coordinate day to day activities? On a given day, who is in charge of a patient/client? How often are the patient reports/information updated? Are these reports/information shared between team members and across settings? What are the challenges of coordinating care *in this service*? Is there something you do *differently* in coordinating care that really helps you do it better that you would like to share?	Refined for Delphi
14.	How frequently do you meet with other team members? What disciplines are present in MDTs?	Refined for Delphi
15.	How are responsibilities shared among team members and how are responsibilities allocated?	Removed
16.	Do you use Electronic Palliative Care Co-ordination Systems (EPaCCS)? If not, how do you keep track of care?	Refined for Delphi
17.	How do patients/clients access your services?	Refined for Delphi
18.	How do you assess patients/clients in terms of their care needs? How do you decide what to prioritize?	Refined for Delphi
19.	Does everyone get an initial assessment of all their needs (e.g. financial advice) –how are people prioritized/flagged?	Removed

20.	What are the services you offer in relations to emergencies?	Refined for Delphi
21.	What services/interventions do you have in terms of managing your client's conditions/issues? E.g. symptom management, pharmacological interventions, psycvhological care, social care	Refined for Delphi
22.	What kind of challenges do your patients experience in carrying on with their day to day activities/activities of daily living? What services do you do to assist them? Eg. Bathing, house work, meal prep, rehab, provision of equipment, transport, spiritual needs of patients	Refined for Delphi
23	What are the services do you offer as your patient's approaches the last days of their life?	Refined for Delphi
24	What services do you provide after a patient dies?	Refined for Delphi
25	What routine bereavement support do you provide? Do you assess bereavement risk – how? Qualitatively or do you use a standardized checklist of risk? If you standardized checklist which one do you use? What bereavement follow up do you have in place for individuals at high risk?	Refined for Delphi
26	What are the activities that you do that:<br>a. are unique to your service?<br>b. important for your service?<br>c. that you would like to do more of or start doing in your service?	Removed
27	What is the one thing that sets your model of care and how you deliver it apart? What are the strengths and limitations?	Removed
28	What are your goals for the future in terms of existing or new models of care?	Removed

Appendix 2: Final sixteen criteria and four contextual criteria

Setting of care

Criterion 1.	Within which setting is this service/team delivered?

	☐ In-patient hospital<br>☐ In-patient hospice<br>☐ Home-based care (including in a patient's own home, nursing or residential care home)<br>☐ Care home/nursing home only<br>☐ Outpatient<br>☐ Day-care<br>☐ Drop-in service<br>☐ Other ambulatory…………………………………………………<br>☐ Prison<br>☐ Other………………………………………………………………

Criterion 2.	What type of care does this service/team provide? (Please tick as many as apply)
	☐ Direct 'hands on' care or procedures<br>☐ Direct advice and support to patients and families<br>☐ Indirect advice and support to other professionals caring for individual patients.<br>☐ Education and training to professionals (not in relation to specific individual patients)

Criterion 3.	How many new referrals and re-referrals are accepted **and seen** annually by this service/team?
	Answer…………………………………………….

Criterion 4.	Which professions are included in this service/team? (Please tick as many as apply)
	☐ Doctors<br>☐ Registered Nurses<br>☐ Health Care Assistants<br>☐ Social Workers<br>☐ Pharmacists<br>☐ Physiotherapists<br>☐ Occupational Therapists<br>☐ Speech & Language Therapists<br>☐ Complementary Therapists<br>☐ Dietitian<br>☐ Psychiatrists<br>☐ Psychologists<br>☐ Counsellors<br>☐ Spiritual Workers/Chaplains<br>☐ Welfare Workers<br>☐ Volunteers<br>☐ Administration<br>☐ Other (please specify) ……………………………………………

44

Criterion 5.	How is care provided by this service/team? (Please tick as many as apply)
	☐ Face to face<br>☐ Telephone advice or support<br>☐ Telecare<br>☐ E-health provision<br>☐ Other (please specify) ……………………………………………………………

45

Criterion 6.	Which of the following does your service provide to your patients? (Please tick as many as apply)
	☐ Sitting service (staying with patients for planned periods of time)<br>☐ Subcutaneous infusions by syringe driver<br>☐ Blood tests<br>☐ Ultrasounds<br>☐ X-rays<br>☐ Other medical imaging<br>☐ Ascites drainage<br>☐ Pain intervention procedures<br>☐ Intravenous fluid infusions<br>☐ Blood transfusions<br>☐ Bisphosphonate infusions<br>☐ Other blood product transfusions<br>☐ Antibiotics<br>☐ Other (please specify) ……………………………………………………………<br>☐ Not applicable

Criterion 7.	Can referrals to this service/team be accepted **and seen** at all times? (24/7)? ?
	☐ Yes<br>☐ No, '24/7 referrals cannot be made to this service<br>☐ Other____

Criterion 8	When is the 'out of hours' care service available? (Please tick as many as apply)
	**<u>Weekdays</u>**<br>☐ Evenings<br>☐ Overnight<br>**<u>Weekends</u>**<br>☐ Daytime<br>☐ Evening<br>☐ Overnight

Criterion 9	Does this service/team provide any 'out of hours' care for patients already known to this service?		
	Medical	Nursing	
	☐	☐	Yes- resident/ on site
	☐	☐	Yes- on-call/ not on site
	☐	☐	No 'out of hours' service (if No, please go straight to Criterion 18)

Criterion 10	How is this 'out of hours' care provided? (Please tick as many as apply)
	☐ Face to face<br>☐ Telephone<br>☐ Other (please specify) …………………………………………….

Criterion 11	Which type of care is provided in this 'out of hours' service?
	☐ 'Hands on' care (direct 'hands on' care or procedures)<br>☐ Advisory care (may include: advice, support or prescribing)<br>☐ Both 'hands on' and advisory care

Criterion 12.	Does this service/team offer education and training to professionals outside your organisation?
	☐ Yes<br>☐ No

Criterion 13	Does this service/team use any standard experience and outcome measures?
	☐ Yes, both experience and outcome measures<br>☐ Just outcome measures<br>☐ Just experience measures<br>☐ No

Criterion 14.	What is the standard follow-up routinely offered to families after death? (Please tick as many as apply)
	☐ Letter<br>☐ Phone<br>☐ Face to face visit/ meeting<br>☐ Group support<br>☐ Other (please specify) ………………………………………………<br>☐ None

67

Criterion 15	Does this service/team offer bereavement care for those at higher risk of complex grief? (Please tick as many as apply)		
	Bereaved Adults	Bereaved Children	
	☐	☐	Yes
	☐	☐	Only standard bereavement care is available
	☐	☐	No bereavement care available

68

Criterion16.	What are the primary diagnoses of those receiving palliative care in your service/team?
	☐ Cancer<br>☐ Non-cancer<br>☐ Any life-limiting illness (Both cancer and non-cancer)<br>☐ Specific diagnoses only (please specify) …………………………………………

69

Contextualised criteria

Criterion 17.	Is your service/team a statutory/public service, a voluntary or charitable service, or other?		
	Managed by	Funded by	
	☐	☐	Statutory/public (i.e. NHS)
	☐	☐	Voluntary/charitable service
	☐	☐	Both statutory/public and voluntary/charitable

	☐ ☐ Other (please specify) ………………………………………………………………

Criterion 18.	Does this service/team accept patient or family self-referrals?
	☐ Yes<br>☐ No

Criterion 19.	Does this service/team have standard criteria for the discharge of patients from this service/team?
	☐ Yes<br>☐ No

Criterion 20.	What is the purpose of the care provided in this service/team? (Please tick as many as apply)
	☐ Symptom control<br>☐ Family and carer support<br>☐ Psychological care<br>☐ Bereavement care<br>☐ Care of the dying (last few days of life)<br>☐ Respite care<br>☐ Spiritual care<br>☐ Rehabilitation<br>☐ Other (please specify) ………………………………………………………………

<u>Appendix 3: *Criterion 4 comparing which disciplines are included in services (0 = no 1= yes 2 = access but not employed by team)*</u>

Service identifier	doctor	RN	HCA	Social worker	pharmacist	physio	OT	Speech therapists	complementary	dietitian	psychiatrist	psychologist	counsellor	chaplain	welfare	admin	volunteers	other
**A**	1	1	0	0	0	0	0	0	0	0	0	0	0	0	0	1	0	0
**B**	0	1	0	0	0	0	0	0	0	0	0	0	0	0	0	1	0	0
**C**	1	1	0	1	0	1	1	0	1	1	0	1	1	1	1	1	1	0
**D**	1	1	1	1	1	1	1	0	1	0	0	0	1	1	0	1	1	0
**E**	1	0	0	0	0	0	0	0	0	0	0	0	0	0	0	1	0	0
**F**	1	1	0	0	0	0	1	0	0	0	0	0	0	0	0	0	0	0
**G**	1	1	0	0	0	0	0	0	0	0	0	0	0	0	0	0	0	0
**H**	1	1	1	1	1	1	1	2	1	1	2	2	1	1	1	1	1	0
**I**	1	1	2	1	2	2	2	0	2	2	0	1	1	2	2	1	2	0
**J**	1	1	2	2	2	2	2	2	0	2	2	2	0	2	0	1	0	0
**K**	1	1	2	1	2	2	2	2	0	2	2	2	0	2	0	1	0	0
**L**	1	1	2	1	2	2	2	2	0	2	2	2	0	2	0	1	0	0
**M**	1	1	2	1	2	2	2	2	2	2	2	1	0	2	0	1	2	0
**N**	1	1	1	1	1	1	1	1	1	1	1	1	1	1	1	1	1	0
**0**	1	1	1	1	1	1	1	1	1	1	0	1	1	1	1	1	1	0
**P**	1	1	1	1	1	1	1	2	1	2	0	0	1	1	0	1	1	1
**Q**	1	1	1	1	1	1	1	1	1	1	1	0	1	1	0	1	1	1
**R**	1	1	1	1	1	1	1	2	1	1	2	2	1	1	1	1	1	1
**S**	1	1	1	0	2	2	1	1	0	2	0	0	0	0	0	1	1	1

Appendix 4: Flowchart of methods

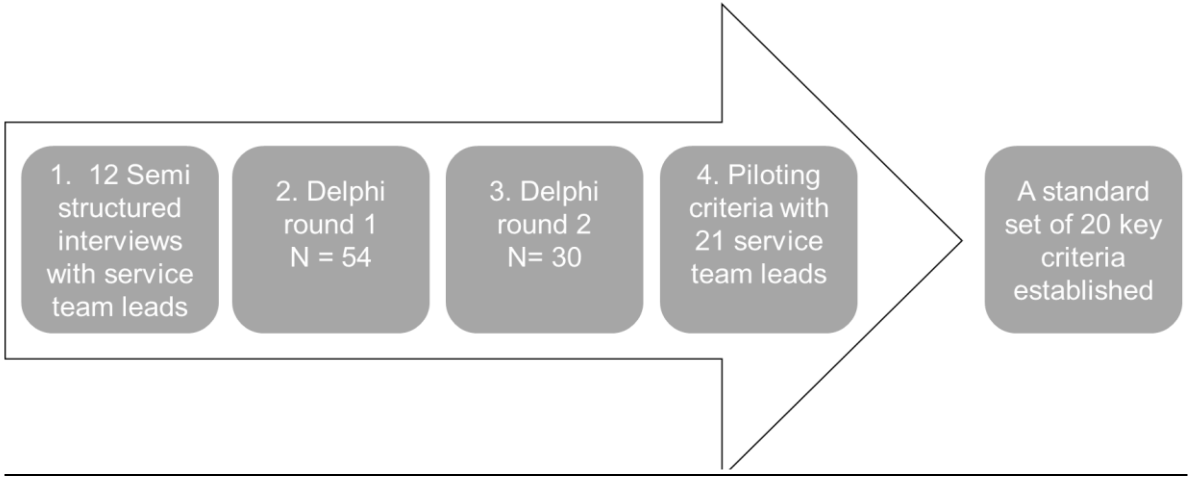

Appendix 5: Rapid Review Strategy

To identify potentially relevant studies for inclusion, the following electronic databases were searched: MEDLINE; EMBASE; CINAHL; PsycINFO; GLOBAL HEALTH and the Cochrane Library. We limited our search from 1st of January 2010 until 31st of June 2015. Following brainstorming of relevant terms, MeSH (Medical Subject Headings) browser to find suitable subject terms related to the search topic. The following search string was used for search 'Delivery of Health care' AND 'palliative care' OR 'end of life care' AND 'Model' NOT 'Education' NOT 'Treatment'. Peer-reviewed articles published in English in Public Health discipline was included. Articles on treatment interventions, and education were excluded. 271 articles were identified. Abstracts and titles were screened for components of models of care. 40 articles described components of different models of care.
